# Evaluation of the Cytotoxicity of Biochar Aqueous Extract in Caco-2 Cells: Time-Dependent Regulation of Apoptosis, Associated with miRNA Modulation

**DOI:** 10.3390/molecules31060989

**Published:** 2026-03-16

**Authors:** Sidra Amin, Klaudia Marcinkowska, Magdalena Wołoszyńska, Sebastian Opaliński, Dawid Skrzypczak, Paweł Wiercik, Łukasz Bobak, Agnieszka Śmieszek

**Affiliations:** 1 Department of Genetics, Faculty of Biology and Animal Sciences, Wroclaw University of Environmental and Life Sciences, ul. Kozuchowska 7, 51-631 Wroclaw, Poland; 2 Laboratory of Preclinical Research “In VetBio”, Department of Pharmacology and Toxicology, Faculty of Veterinary Medicine, Wroclaw University of Environmental and Life Sciences, Norwida 31, 50-375 Wroclaw, Poland; 3 Department of Cellular Molecular Biology, Faculty of Biotechnology, University of Wroclaw, Joliot-Curie 14a, 50-383 Wroclaw, Poland; 4 Department of Environment Hygiene and Animal Welfare, Faculty of Biology and Animal Sciences, Wroclaw University of Environmental and Life Sciences, Chelmonskiego 38C, 51-630 Wroclaw, Poland; 5 Department of Advanced Material Technologies, Faculty of Chemistry, Wroclaw University of Science and Technology, Smoluchowskiego 25, 50-370 Wroclaw, Poland; 6 Institute of Environmental Engineering, Faculty of Environmental Engineering and Geodesy, Wroclaw University of Environmental and Life Sciences, Grunwaldzki Square 24, 50-363 Wroclaw, Poland; 7 Department of Animal Products Technology and Quality Management, Faculty of Biotechnology and Food Science, Wroclaw University of Environmental and Life Sciences, Chelmonskiego 37, 51-630 Wroclaw, Poland; lukasz.bobak@upwr.edu.pl

**Keywords:** biochar, intestinal cell model, viability regulation, time-dependent cellular responses

## Abstract

Biochar, a carbon-rich material traditionally used to improve soil health and as a feed additive, has recently attracted attention for its potential biological activity. This study examined the effects of an aqueous biochar extract (BC-AE) on human intestinal epithelial cells (Caco-2), focusing on its influence on cell viability and apoptosis. The metabolic activity of Caco-2 cells exposed to BC-AE was first evaluated using an MTS assay. A concentration of 3 mg/mL, which promoted Caco-2 metabolic activity, was selected for further testing at 24 and 72 h. The effect of BC-AE on cell viability was assessed by epifluorescence microscopy (morphology) and flow cytometry (apoptosis profiling). The transcriptional response of cell viability-related genes (*BAX*, *BAD*, *BCL-2*, *BCL-xL*, *MCL-1*, *P21*, and *P53*) and microRNAs (miR-15b, miR-19, miR-21, miR-33a, miR-155, and miR-486) was analyzed by RT-qPCR. In parallel, selected proteins (BAD, BAX, BCL-2, and MCL-1) were examined by Western blotting. We showed that BC-AE decreased cell viability after 24 h via late apoptosis, while 72 h exposure increased necrosis without further viability loss. Both BAX and MCL-1 protein levels increased in Caco-2 cells after 72 h of BC-AE treatment, and miR-15b and miR-21 were upregulated, suggesting the involvement of a regulatory mechanism controlling cell survival. The obtained findings highlight the importance of considering both concentration and exposure duration when assessing biochar bioactivity and represent an additional contribution to the ongoing effort to better understand the biological role.

## 1. Introduction

Biochar is a carbon-rich material obtained from the pyrolysis of organic biomass, whether of plant or animal origin. It has gained recognition as a functional feed additive in livestock production [1,2]. Its purported benefits include improved gut health, enhanced nutrient absorption, and toxin adsorption, making it a promising candidate for biomedical applications [1,3]. Despite the growing use of biochar, its biological effects remain poorly understood, as well as its influence on proliferation and survival pathways. Although recent data, including the study by Pinelli et al. [4], indicate that biochar may affect mammalian cells, available evidence remains limited and concerns mainly particulate biochar, whereas the biological effects of soluble biochar-derived components in intestinal epithelial models remain insufficiently characterized. Emerging evidence suggests that biochar may influence molecular pathways involved in cellular homeostasis [5,6], but its role in regulating non-coding RNAs (ncRNAs) remains undetermined.

MicroRNAs (miRNAs), small non-coding RNAs approximately 20–24 nucleotides in length, comprise a significant class of regulatory RNAs that play essential roles in controlling gene expression and modulating various cellular functions, including proliferation, viability, and differentiation [7]. Moreover, the class of microRNAs responsive to dietary components that can be transferred through food intake has been recognized as an essential mediator of nutritional regulation, due to its involvement in intestinal homeostasis and cellular metabolic adaptation [8]. At the same time, the concept of microRNAs being transferred through food intake remains controversial, as their stability during digestion, uptake by intestinal cells, and biological relevance in mammals are still under debate, suggesting that limited stability and uncertain bioavailability may substantially limit their functional relevance [9,10]. In this broader context, it is also important to consider whether biochar-derived soluble components may influence the endogenous microRNA network involved in intestinal epithelial cell responses.

Indeed, the role of regulatory microRNAs in mediating cellular responses to biochar exposure remains largely unexplored, underscoring the need for in-depth mechanistic investigations [11]. Addressing these knowledge gaps would be crucial for determining the safety and biological relevance of biochar in various contexts, including biomedical applications such as antimicrobial and anticancer strategies, drug delivery systems, and biosensing platforms. This is particularly important for orally administered or gastrointestinally exposed biochar-based materials, for which intestinal epithelial models such as Caco-2 are relevant to evaluate cytotoxicity, barrier integrity, and absorption-related effects. Biochar is also used in livestock production as a feed additive, particularly in poultry, cattle, and swine [2,12].

The Caco-2 cell line, widely recognized as a robust in vitro model of the human intestinal barrier, provides a valuable platform for investigating biochar influence at cellular and molecular levels [13]. The cell line is derived from human colorectal adenocarcinoma. Although this is a cancer cell line, it is applied for nutrient absorption studies and cellular metabolism analyses focused on mimicking the physiological functions of the intestinal epithelium [13,14]. Its ability to differentiate into polarized, enterocyte-like cells makes it a reliable model for investigating intestinal barrier function, transport mechanisms, and the impact of bioactive compounds on gut health [15]. While Caco-2 has been successfully used to study bioactive molecules and food additives such as curcumin [16], resveratrol [17], and quercetin [18], its application in evaluating biochar remains largely unexplored.

In this study, we hypothesized that soluble biochar-derived components may modulate intestinal epithelial cell responses by influencing apoptosis- and survival-related pathways, including selected regulatory microRNAs. To test this hypothesis, we evaluated the biological activity of an aqueous biochar extract (BC-AE) in the Caco-2 model by analyzing metabolic activity, apoptosis dynamics, and the expression of apoptosis-related genes and selected microRNAs, i.e., miR-15b-5p, miR-19a-3p, and miR-21-5p, due to their established roles in the regulation of cell survival and proliferation, as well as their potential relevance to nutrition-related biological effects [19,20,21].

In this context, evaluating how biochar-derived compounds affect fundamental cellular functions, such as viability, metabolism, and gene expression, is vital, given the current gap in knowledge about their direct biological effects at the cellular and molecular levels. Importantly, our findings support the concept that biochar may exert regulatory effects beyond its physical and chemical properties, thereby providing new insight into its bioactivity and advancing our understanding of its potential applications in animal and human health.

## 2. Results

### 2.1. Biochar and BC-AE Characterization

Biochar is a heterogeneous carbonaceous material rather than a discrete molecular entity, so the study does not report a previously undescribed pure compound. However, comprehensive material characterization, including FTIR and elemental analysis, CHNS/O, ash content, and BET surface area, was performed.

The elemental composition of the biochar sampled, determined by CHNSO analysis, was (wt.%): C 84.8, H 2.21, N 0.730, S 0.433, and O 7.79. The mean ash content (AC) was 4.52%. Detailed results for the concentrations of other elements in biochar and the BC-AE samples, including potentially toxic As, Cd, and Pb, are presented in Table S1. The BET and Density Functional Theory (DFT) surface areas of biochar samples were on the level of 412 and 464 m^2^⋅g^−1^, respectively [22].

Given that the FTIR spectrum of the BC-AE samples was dominated by water bands (3285 and 1636 cm^−1^), FTIR measurements were additionally performed on the parent biochar to characterize functional groups in the solid material from which the extract was prepared. The lack of intense peaks in the entire spectral range (Figure S2) indicates high aromatization of the material as a result of pyrolysis at 600 °C [23].

At temperatures above 500 °C, oxygenated groups disappear, i.e., O-H from cellulose and lignin in the region of 3500–3000 cm^−1^, or C–O groups around 1700 cm^−1^ [24]. Hence, the spectrum shows no peaks in this wavenumber range. Minor sharp features observed in the 2300–1900 cm^−1^ region and at around 1700 cm^−1^ were within the noise level (≤0.005 absorbance units) and were not assigned to specific functional groups. Peak at 1560 cm^−1^ can be attributed to the vibrations of C=C and C=O bonds in aromatic compounds [23]. In the 2010 research by Keiluweit et al. [24] it can be observed that a distinct peak in the 1030 cm^−1^ region, corresponding to C–O vibrations in cellulose, hemicellulose, and lignin, decreases with increasing temperature and becomes significantly flattened in the spectrum of the biochar sample obtained from grass at temperatures above 400 °C. Therefore, the broad peak, whose flat top extends from about 1150 to 1000 cm^−1^, can be assigned to the vibrations of the C–O group in lignin- and cellulose-derived transformation products. Signals in this region may also arise from the C–H deformation of cellulose-derived substituted aromatics [24]. The peaks at 871, 799, and 744 cm^−1^ correspond to out-of-plane deformations of aromatic C–H [24].

Although FTIR indicated a highly condensed aromatic carbon matrix, the technique is not sufficiently specific to evaluate potentially hazardous low-molecular-weight by-products. Because polycyclic aromatic hydrocarbons (PAHs) can be generated during biomass pyrolysis and may remain associated with biochar surfaces and pores—with the potential to partition into aqueous extracts—targeted PAH analysis was conducted. PAHs were assessed in both the aqueous biochar extracts and in acetonitrile extracts obtained after 72 h of extraction of the biochar. No PAHs were detected in either extract type; all analytes were below the limit of detection (LOD = 0.5 µg/kg).

### 2.2. BC-AE Shows Low Cytotoxicity Toward Caco-2 Cells in a Screening Assay

**Figure 1:**
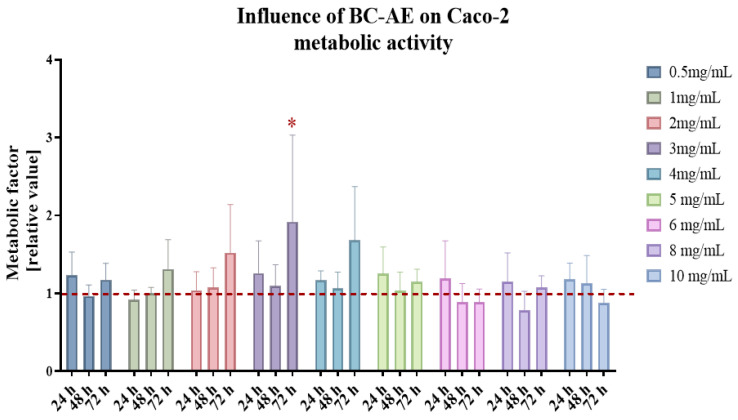
The effect of BC-AE on the metabolism of human colorectal adenocarcinoma cells, Caco-2. Cellular metabolism was assessed using the MTS assay. Metabolic activity of Caco-2 cells exposed to biochar, expressed as a metabolic factor relative to untreated control cultures; the reference value of 1 is indicated by a red dashed line. A comparative analysis was conducted using results from three independent experiments, each performed in technical triplicate. The data are displayed as bar graphs, representing the mean ± standard deviation (SD). Statistical significance is denoted by asterisks, where * indicates *p*-value < 0.05.

An MTS assay was performed on Caco-2 cells to evaluate the cytotoxicity and metabolic effects of varying BC-AE concentrations (Figure 1). Cell viability and metabolic activity were monitored over time. Analysis of BC-AE’s influence on the metabolic activity of Caco-2 cells showed its low cytotoxicity in comparison with untreated control cultures. No dose- and time-dependent response was observed. The BC-AE improved the metabolism of Caco-2 cells at doses of 2-, 3-, and 4 mg/mL. The effect was noted after 72 h. Nevertheless, a statistically significant increase in metabolic activity, as compared with untreated control cultures, was observed only in cultures treated with 3 mg/mL of BC-AE (Figure 1). Consequently, this concentration was selected for subsequent functional assays.

### 2.3. Functional Assays—Analysis of Effects of a 3 mg/mL Biochar Aqueous Extract (BC-AE) on Caco-2 Activity

#### 2.3.1. BC-AE Maintains Caco-2 Cell Morphology and Structure, but Long-Term Treatment May Gradually Impact Their Growth Pattern

**Figure 2:**
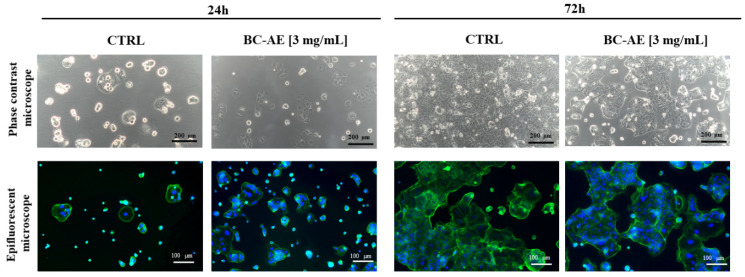
The effect of BC-AE prepared from a 3 mg/mL sample on Caco-2 cell morphology and growth pattern. The nuclei were stained with DAPI (blue signal), and the cytoskeleton was stained with phalloidin-488 Atto (green signal). Caco-2 cells were treated with BC-AE and observed at designated time points to assess potential morphological alterations and changes in culture dynamics. Analyses were conducted using an inverted phase-contrast microscope to evaluate the overall monolayer structure and cell density, as well as epifluorescence microscopy to visualize cellular details. The experiment was repeated three times independently (biological replicates), with three parallel cultures per condition in each experiment (technical replicates). For each culture, three microscopic fields were recorded. The representative images illustrate the impact of BC-AE on cell shape, colony formation, and spatial organization within the monolayer. Observations and interpretations are further discussed in the main text. Scale bars are provided within each image.

Cell morphology was monitored using both phase contrast and epifluorescence microscopy to assess structural integrity and growth patterns under the influence of BC-AE at a concentration of 3 mg/mL (Figure 2). The morphology of Caco-2 cells was characteristic, typical of epithelial-origin cell lines, with a polygonal shape in control cultures. After 24 h of BC-AE treatment, no significant morphological alterations were observed in Caco-2 cultures. Interestingly, BC-AE-treated colonies appeared more developed than those of the untreated controls, suggesting a possible stimulatory effect during early exposure.

After 72 h of treatment, distinct changes in overall culture appearance and growth dynamics were observed. Treated cells failed to establish a continuous monolayer, and cell colony expansion was noticeably reduced compared to the control group. Despite these alterations, no overt signs of cytotoxicity, such as cell shrinkage, detachment, or membrane blebbing, were detected.

Microscopic observations suggest that prolonged exposure to BC-AE may exert a mild inhibitory effect on cell proliferation or colony formation (Figure 2). Overall, the data indicate that while BC-AE does not disrupt the structural morphology or integrity of Caco-2 cells, it may influence their growth dynamics over time.

#### 2.3.2. BC-AE Temporarily Regulates the Dynamics of Apoptosis and Necrosis in Caco-2 Cells

**Figure 3:**
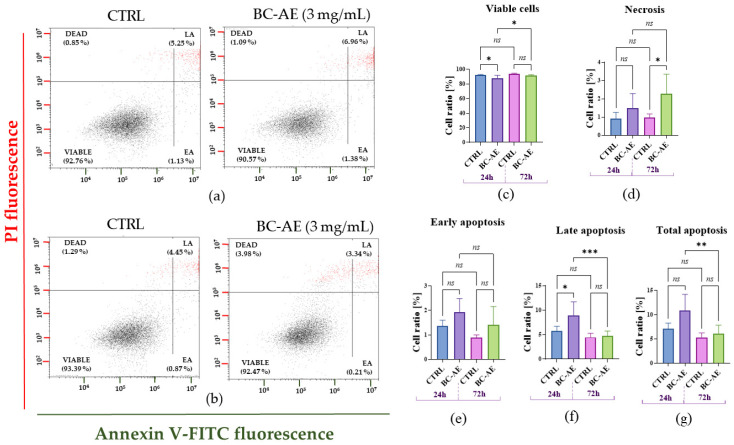
The distribution of Caco-2 cells based on Annexin V and propidium iodide (PI) staining was assessed using flow cytometry. The representative dot-blots are presented for measurements made after 24 h (**a**) and 72 h (**b**). Viable cells are located in the Annexin V–/PI– quadrant (left bottom square). Early apoptotic (EA) cells are Annexin V+/PI–, indicating externalization of phosphatidylserine without membrane permeabilization, and are shown in the right bottom gate. Late apoptotic (LA) or secondary necrotic cells are Annexin V+/PI+ (upper right gate), while necrotic cells (dead) are Annexin V–/PI+ (upper left gate). A comparative analysis was conducted using results from three independent experiments, each performed in technical triplicate. The comparative analysis revealed differences between treated and untreated cells at various time points (24 and 72 h) in terms of Caco-2 distribution, including viable cells (**c**), necrotic cells (**d**), early apoptosis (**e**), late apoptosis (**f**), and total cell apoptosis (**g**). Means ± SD. * *p*-value < 0.05, while the ns symbol refers to non-significant differences. Statistically significant differences were indicated as follows: * *p*-value < 0.05; ** *p*-value < 0.01, and *** *p*-value < 0.001, while non-significant differences are denoted as ns.

Annexin V/PI staining was used to assess the impact of BC-AE (3 mg/mL) on Caco-2 cell viability, thereby distinguishing between apoptotic and necrotic cells (Figure 3). The viability of Caco-2 cells after 24 h treatment with BC-AE decreased, which was associated with an increase in late apoptotic cells (Figure 3a,c–g). No noticeable effect on cell viability was observed for cultures treated with BC-AE for 72 h, and apoptosis was not induced. Nonetheless, in those cultures, a significant increase in the population of necrotic cells was noticed (Figure 3b–g).

#### 2.3.3. BC-AE Modulates the Endogenous Levels of Survival-Related miRNAs

**Figure 4:**
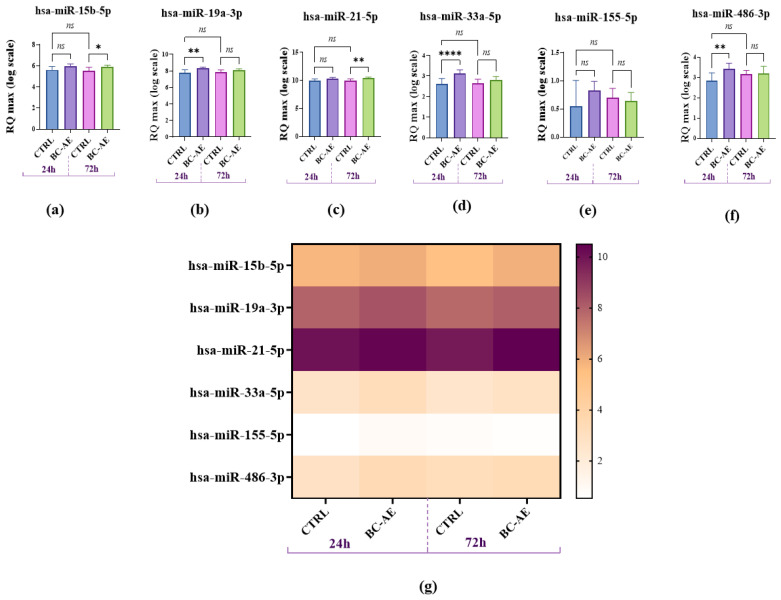
Effect of BC-AE (3 mg/mL) on the relative expression (RQ) of selected miRNAs in Caco-2 cells after 24 h and 72 h of exposure. (**a**) hsa-miR-15b-5p expression; (**b**) hsa-miR-19a-3p expression; (**c**) hsa-miR-21-5p expression; (**d**) hsa-miR-33a-5p expression; (**e**) hsa-miR-155-5p expression; (**f**) hsa-miR-486-3p expression; (**g**) heat map summarizing the expression patterns of all analyzed miRNAs across the experimental groups. The study was performed in three independent biological replicates, each with three technical replicates per condition. Among the analyzed miRNAs, hsa-miR-21-5p showed the highest expression, whereas hsa-miR-155-5p showed the lowest expression. Statistically significant differences were indicated as follows: * *p*-value < 0.05; ** *p*-value < 0.01 and **** indicates *p* < 0.0001, while non-significant differences are denoted as ns.

To assess the impact of BC-AE on endogenous expression of selected (based on literature) miRNAs in Caco-2 cells, RT-qPCR analysis was performed following 24- and 72 h treatments. This approach enabled the evaluation of the temporal dynamics and specificity of miRNA regulation. RT-qPCR measurement of endogenously accumulated miRNAs showed that BC-AE significantly increases the levels of miR-19a, miR-33a-5p, and miR-486-3p after 24 h (Figure 4b,d,f, respectively). The levels of miR-15 b-5p and miR-21-5p were not affected by BC-AE treatment after 24 h (Figure 4a,c). In cultures treated for 72 h, the levels of miR-15b-5p and miR-21-5p were significantly upregulated (Figure 4a,c), while miR-19a-3p, miR-33a-5p, and miR-486-3p did not alter significantly (Figure 4b,d,f). The levels of miR-155-5p were not changed by BC-AE treatment at the analyzed time points (Figure 4e). The highest accumulation of miRNA transcripts in Caco-2 cells was observed for miR-21-5p, while the lowest expression was noted for miR-155-5p, as indicated in the heat map (Figure 4g).

#### 2.3.4. BC-AE Modulates Anti- and Pro-Apoptotic Signals at the mRNA and Protein Levels

**Figure 5:**
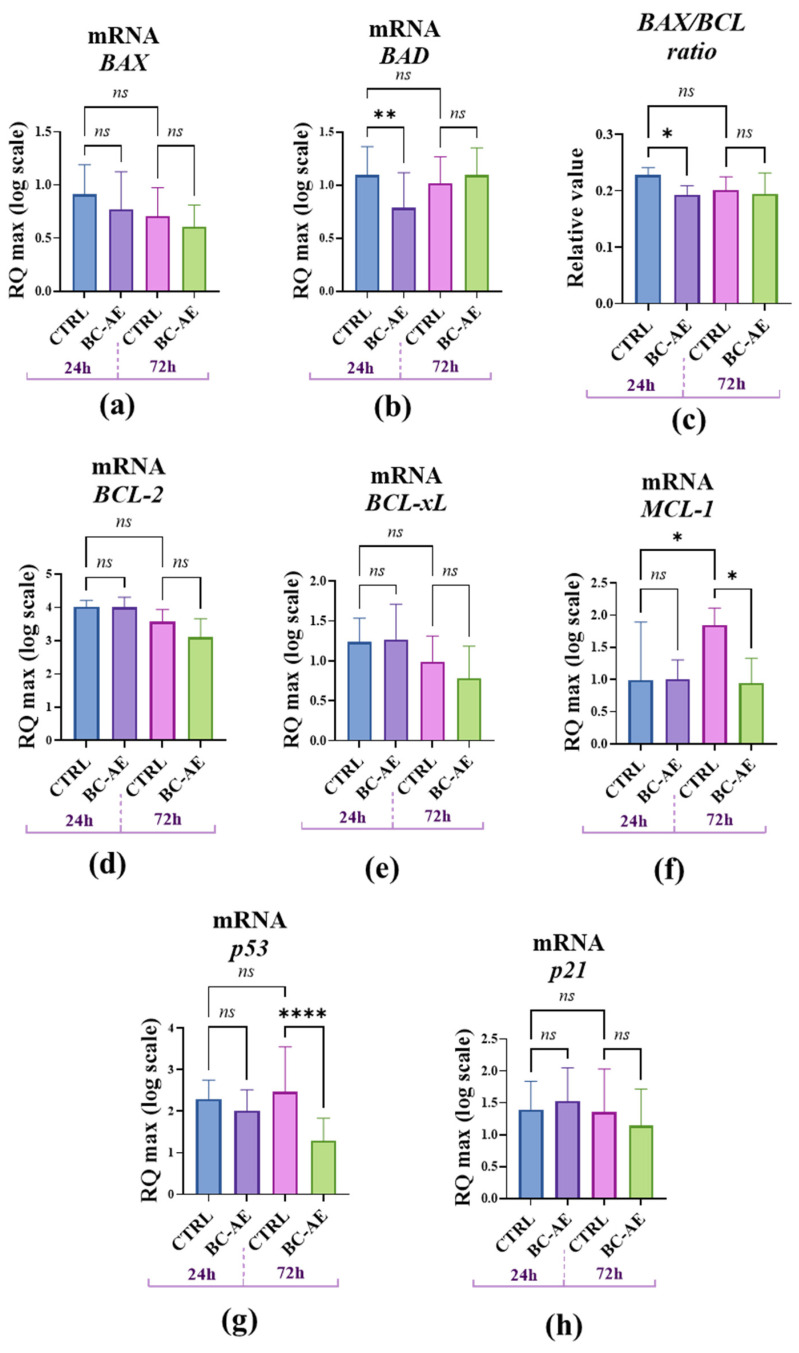
The influence of BC-AE treatment on the expression of key apoptosis- and cell cycle-regulating genes in Caco-2 cells (3 mg/mL, 24 h and 72 h). The analysis included the determination of pro-apoptotic markers, i.e., *BAX* and *BAD* (**a**,**b**), and anti-apoptotic markers, i.e., *BCL-2* (**d**), *BCL-xL* (**e**), and *MCL-1* (**f**). Measurements were performed in three independent biological replicates, each with three technical replicates per condition. The BAX/BCL-2 ratio was determined to assess the balance between pro- and anti-apoptotic signaling (**c**). The mRNA levels of *P53* (**g**) and *P21* (**h**) were analyzed to evaluate the activation of cell cycle regulatory pathways. A comparative analysis was conducted to evaluate the significance of the observed expression patterns. Statistically significant differences were indicated as follows: * *p*-value < 0.05; ** *p*-value < 0.01 and **** indicates *p* < 0.0001, while nonsignificant differences are denoted as ns.

**Figure 6:**
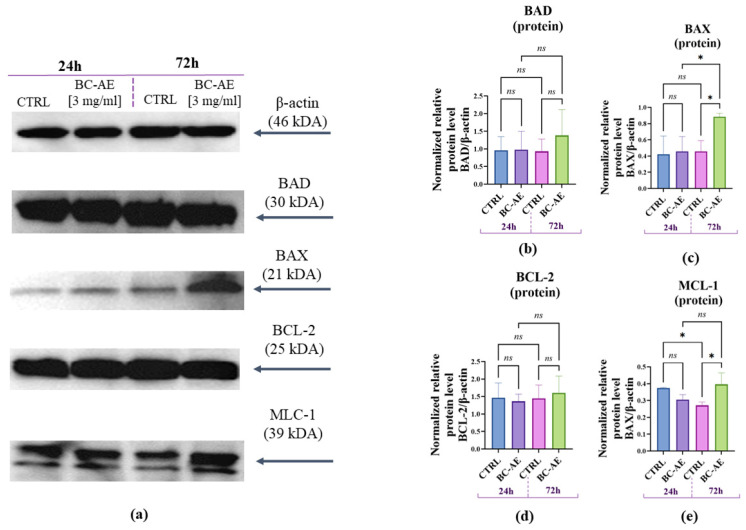
The influence of BC-AE treatment on the expression of key apoptosis-related proteins in Caco-2 cells (3 mg/mL, 24 h and 72 h). The experiment was performed in three independent biological replicates, each with three technical replicates per condition. Western blot analysis was performed to determine the protein expression levels of pro-apoptotic markers BAX and BAD (**a**,**b**,**d**), and anti-apoptotic markers BCL-2 and MCL-1 (**a**,**c**,**e**). β-actin was used as a reference molecule. Quantitative analysis was conducted to evaluate the relative protein levels normalized to β-actin. A comparative analysis was conducted to evaluate the significance of the observed changes at both time points * *p*-value < 0.05; while nonsignificant differences are denoted as ns.

To further explore the molecular mechanisms underlying the observed effects of BC-AE on cell viability, we investigated its impact on mRNA expression of common biomarkers associated with cellular viability using RT-qPCR at two time points. Gene expression analysis of well-known factors related to cell survival regulation revealed that pro-apoptotic *BAX* and anti-apoptotic *BCL-2* are not directly affected by BC-AE treatment (Figure 5a,d). However, the relative accumulation of BAX/BCL-2 mRNAs was significantly decreased (Figure 6c), suggesting potential activation of anti-apoptotic signals. This effect, noted after 24 h, is also associated with a significant decrease in mRNA levels for the pro-apoptotic Bcl-2-associated death protein, i.e., *BAD* molecule (Figure 5b). Transcript levels of the anti-apoptotic *BCL-xL*, *MCL-1*, and *P21* genes were not significantly changed after 24 h (Figure 5e,f,h). The mRNA levels of all tested genes remained unchanged after 72 h of treatment, except for *MCL-1* and *P53*, whose levels decreased significantly (Figure 5f,g).

To investigate the molecular pathways through which BC-AE influences Caco-2 viability, Western blot analysis was used to examine the expression of key apoptosis-related proteins at 24 and 72 h following BC-AE treatment (Figure 6). BC-AE did not affect the intracellular accumulation of BAD, BAX, BCL-2, or MCL-1 proteins after 24 h of treatment (Figure 6b,e). However, after 72 h, a significant increase in the expression levels of both pro-apoptotic BAX (Figure 6c) and anti-apoptotic MCL-1 (Figure 6e) proteins was detected, suggesting a time-dependent regulatory effect on apoptotic signaling pathways. Interestingly, a decrease in MCL-1 expression was observed in the control cultures over time, which may reflect natural regulatory processes or cellular stress responses occurring independently of BC-AE treatment (Figure 6e).

## 3. Discussion

Biochar is a carbon-rich material increasingly used in sustainable agriculture, for example, as a dietary supplement in poultry production, due to its capacity to enhance gut health and nutrient absorption [2,25,26]. The present study contributes to a better understanding of the biological activity of biochar-aqueous extract (BC-AE), indicating its modulatory effects on cellular metabolism and apoptosis-related signaling pathways. We showed that biochar exhibits low cytotoxicity toward Caco-2 cells, but its biological effects may result from time-dependent interactions with cellular processes, including the initial stimulation of metabolism and maintenance of morphology.

The well-known biochar’s functional role is related to its high porosity and adsorption capacity as well as its ability to modulate the gut microbiome, which may impact nutrient absorption and immune function [2,27]. In the current study, we aimed to explore the bioactive potential of biochar beyond its physical and chemical properties, focusing on its role in modulating the expression of pro-apoptotic and anti-apoptotic genes at the mRNA and protein level. We also explored biochar as a potential regulator of miRNA levels.

Our screening assay indicated that biochar has low cytotoxicity and, at a concentration of 3 mg/mL, may enhance cellular metabolism. This observation aligns with previous studies where biochar displayed minimal cytotoxic effects across various in vitro models, even at relatively high concentrations. An in vitro study using NIH 3T3 mouse fibroblasts, performed by Sigmund et al. (2017), demonstrated that biochar induces a concentration-dependent cytotoxic response, with EC10 values decreasing over time, indicating an increase in toxicity with prolonged exposure [28]. Our results are also consistent with research on plant-derived extracellular vesicles and RNA-containing molecules, which have been shown to modulate host cellular functions [29].

The studies focusing on the molecular mechanisms of biochar action are scarce. Thus, our study further concentrated on functional assays designed to explore how BC-AE interacts with key regulatory apoptotic proteins. We found that BC-AE not only affects cell metabolism but also influences intracellular signaling at the epigenetic level. Specifically, BC-AE modulated the expression of apoptosis-regulating genes, most notably reducing the BAX/BCL-2 ratio and downregulating BAD expression at 24 h, suggesting a shift toward an anti-apoptotic state. These effects were accompanied by increased expression of survival-related miRNAs, such as miR-19a, miR-33a, and miR-486, which may further contribute to enhanced cell viability by promoting pro-survival signaling pathways [30]. Notably, miR-486 also plays an oncosuppressive role in colorectal cancer [31]; therefore, the present results suggest that BC-AE may have biological relevance in the regulation of apoptosis- and proliferation-related pathways in Caco-2 cells, which warrants further investigation also in the context of anticancer features.

Actually, while short-term exposure (24 h) promoted cellular metabolism and did not alter Caco-2 morphology, prolonged exposure (72 h) led to significant changes in the cellular architecture of Caco-2, characterized by disrupted monolayer formation compared to the control culture. The obtained data are consistent with the findings of Sigmund et al., who demonstrated that biochar cytotoxicity may be linked to its effects on cell morphology. However, in their study, this effect was strictly dependent on the concentration of biochar and became apparent only after 48 h of exposure [28].

The sustained presence of BC-AE may shift the cellular balance toward apoptosis and necrosis, possibly due to cumulative cellular stress and a miRNA-regulated network. At 72 h, there was a marked upregulation of miR-15b and miR-21, both of which are known to regulate apoptosis [32,33]. The miR-15/16 family is known for its pro-apoptotic role, primarily through the downregulation of anti-apoptotic genes, such as *BCL-2* [32]. At the same time, miR-15b-5p has been shown to function as a tumor suppressor in hepatocellular carcinoma (HCC) by promoting endoplasmic reticulum stress, inducing apoptosis, and inhibiting cell proliferation through the downregulation of its target gene, Rab1 [34].

Notably, miR-21, also regulated by BC-AE, is well-documented for its role in cell proliferation and survival [32]. In particular, it has been shown to suppress the viability, migration, and invasion of esophageal cancer cells, thereby enabling their apoptosis by inhibiting the PI3K/AKT signaling pathway, a key regulator of cell survival and proliferation [35]. On the other hand, miR-21, often described as an oncogenic microRNA (oncomiR), is known to be upregulated in colorectal cancer and associated with tumor aggressiveness, promoting cell survival and proliferation by targeting tumor suppressor genes and modulating apoptosis-related proteins [36]. Consequently, miR-21 has context-dependent roles that may tilt the balance toward apoptosis under certain stress conditions [37,38].

Regarding protein expression, although mRNA analysis did not reveal significant transcriptional upregulation of apoptosis-related genes, we observed increased protein levels of BAX and MCL-1 at 72 h. The apparent discordance between mRNA and protein expression is biologically plausible, as transcript levels alone do not fully determine protein abundance. Instead, protein output emerges from the integration of multiple regulatory processes operating beyond transcription, including miRNA-dependent post-transcriptional control, differential translational efficiency, protein degradation, and the temporal kinetics of stress adaptation [39,40]. In this context, the BC-AE-induced modulation of regulatory miRNAs further supports the interpretation that the observed cellular response is governed by multi-level regulatory mechanisms rather than by a simple transcription-to-protein relationship.

This finding suggests that, similarly to miRNA factors, proteins involved in pro- and antiapoptotic pathways also undergo complex modulation under the BC-AE treatment. According to some reports, MCL-1 blocks the progression of apoptosis by binding and sequestering the pro-apoptotic proteins, including BAX, which are capable of forming pores in the mitochondrial membrane [41]. Cytochrome c, released this way into the cytoplasm, induces the activation of caspases, which largely contribute to the macromolecular degradation observed during apoptosis. Inhibition of the pro-apoptotic BAX function by anti-apoptotic MCL-1 has also been reported by other authors, although direct interaction between the two proteins was not required in this case [42]. Given that BAX is a pro-apoptotic protein that promotes cell death, while MCL-1 is an anti-apoptotic protein that supports cell survival, their concurrent upregulation suggests a complex and potentially compensatory cellular response to stress or damage, possibly reflecting the interplay between pro-apoptotic (e.g., miR-15 b-mediated) and pro-survival (e.g., miR-21-mediated) signaling pathways under BC-AE exposure. Additionally, while BAX upregulation is typically associated with apoptosis, its excessive or dysregulated activation in the absence of effective apoptotic execution can promote necrotic cells [43], as observed in Caco-2 cultures after 72 h of treatment. Concurrently, elevated MCL-1 expression may represent a compensatory survival response, also shifting the balance from apoptosis toward necrosis [44].

The obtained findings highlight the dynamic and time-dependent nature of BC-AE’s biological activity, underscoring the need to consider both concentration and exposure duration when evaluating its safety and potential therapeutic applications. Our study demonstrates that BC-AE modulates functional miRNAs that can regulate cellular metabolism and apoptosis in Caco-2 cells. We showed that short-term exposure enhances metabolic activity, supports proper cell morphology, but increases the occurrence of apoptosis. In contrast, prolonged exposure leads to necrosis accompanied by the upregulation of pro-apoptotic microRNAs and apoptosis-related proteins.

Moreover, the regulation of so-called miRs driven by BC-AE may also suggest a novel pathway through which biochar-based products might interact with host cells, influencing gene expression and cellular responses.

While our findings point to these intriguing molecular interactions, they represent only an initial step toward understanding the complex bioactivity of BC-AE and its mechanism of action. A limitation of the present study is that functional assays were performed using a single selected BC-AE concentration. Moreover, although Caco-2 cells represent a relevant intestinal epithelial model, the present study did not include barrier-function analyses; therefore, the findings and observed effects should be interpreted as preliminary and limited to cellular and molecular responses. Further studies are essential to fully unravel the underlying mechanisms and determine biochar relevance in more physiologically relevant models. However, our study is the first attempt to understand the multifaceted nature of BC-AE’s bioactivity and reveals it in a model of human intestinal epithelial cells (Caco-2).

## 4. Materials and Methods

### 4.1. Cell Line

The Caco-2 cell line, derived from human colon carcinoma, was acquired from the American Type Culture Collection (ATCC; HTB-37TM). Cells used for the experiment were established at the 12th passage. Caco-2 cells were treated at 70–80% confluence to ensure a standardized, metabolically active, subconfluent cell population and to avoid the post-confluent differentiation typical of this cell line. Previously developed protocols were employed for culture propagation [45]. In detail, Caco-2 cells were cultured and grown in a 75 cm^2^ flask at 37 °C in an incubator with 95% humidity and 5% CO_2_. The basal growth medium, Eagle’s Minimum Essential Medium (EMEM), was supplemented with 10% fetal bovine serum (FBS) and 1% antibiotics penicillin/streptomycin solution (Sigma Aldrich, Munich, Germany). The complete growth medium (CGM) was changed in cultures every two days. The passage was performed when cells reached approximately 80–90% confluence, and the procedure was carried out using a trypsin solution (TrypLE^TM^ Express; Thermo Fisher Scientific, Warszawa, Poland) according to the manufacturer’s instructions.

### 4.2. Preparation of Biochar Aqueous Extracts (BC-AE)

The biochar used in our experiment was produced from beech and oak wood chips. The chips were ground, sieved, and then dried for 24 h at a temperature of 105 °C. The resulting material was subsequently pyrolysed in a muffle furnace at 600 °C for 240 min. A detailed characterization of the investigated biochar was described by us previously [22,23].

The Biochar Aqueous Extract (BC-AE) was prepared using the method described by Martínez-Gómez et al. [46]. Twenty-four hours before the experiment, biochar was soaked in a complete culture medium for in vitro assays. The stock solutions were prepared at a concentration of 10 mg/mL. The mixture was shaken continuously in the shaking incubator at 100 rpm and incubated overnight at 20 °C. The obtained mixture was centrifuged for 15 min at 4000× *g*, and the supernatant was filtered through a 0.22 μm syringe filter. The aqueous extract obtained during the filtration process was used directly in the assays.

### 4.3. Multielemental Analysis

Multielemental analysis of biochar and extract was performed using the ICP-OES technique—VISTA-MPX spectrometer (Varian, Mulgrave, Australia). Before analysis, samples were subjected to microwave digestion on a START D system (Milestone, Sorisole, Italy) in an adapted time-temperature program. The extract (0.5 g) was mineralized using 2.5 mL of 69% nitric acid(V) (Tracepure, Merck KGaA, Darmstadt, Germany) and 7.5 mL of 36% hydrochloric acid (Tracepure, Merck KGaA, Germany). Biochar (0.1 g) was mineralized in a two-stage program using (1) 3 mL of 96% sulfuric acid (VI) and 3 mL of ultrapure water, and (2) 5 mL of 69% nitric acid(V). The cooled samples were diluted to 50 g. Before the analysis, the spectrometer was calibrated using reference material (27-element standard 100 mg/mL, Ultra Scientific, North Kingstown, RI, USA) [47]. The major elements C, H, N, and S of biochar samples were measured in triplicate using a CHNS analyzer (PerkinElmer, 2400 CHNS/O Series II, Waltham, MA, USA).

### 4.4. Fourier Transform Infrared Analyses

The biochar sample was analysed in the Chemical Laboratory of Wrocław University of Environmental and Life Sciences, where Attenuated Total Reflection-Fourier Transform Infrared (ATR-FTIR) analyses were performed using the Nicolet iN10 integrated infrared microscope with the external Nicolet iZ10 FT-IR module (Thermo Fischer Scientific, Waltham, MA, USA). Each spectrum represented the average of 32 scans in the 400–4000 cm^−1^ wavenumber range at a spectral resolution of 4 cm^−1^.

### 4.5. PAHs Analyses

The determination of selected polycyclic aromatic hydrocarbons (PAHs), namely chrysene, benz(a)anthracene, benzo(b)fluoranthene, and benzo(a)pyrene, in the BC-AE and biochar sample was performed using high-performance liquid chromatography coupled with fluorescence detection (HPLC–FLD). Chromatographic analyses were carried out using an HPLC system (Infinity 1260, Agilent Technologies, Santa Clara, CA, USA) equipped with a binary pump, autosampler, column thermostat, and fluorescence detector. Separation was achieved on a Zorbax Eclipse PAH column (4.6 × 250 mm, 5 µm particle size). The column temperature was maintained at 25 °C. The mobile phase consisted of water and acetonitrile (ACN) applied in a gradient elution mode. At the initial time (0 min), the mobile phase composition was 60% water and 40% ACN, with a flow rate of 2.0 mL/min. The ACN proportion was increased linearly to 100% over 17 min. Fluorescence detection was performed using compound-specific excitation and emission wavelengths as follows: chrysene (excitation 266 nm, emission 408 nm), benz(a)anthracene (excitation 286 nm, emission 408 nm), benzo(b)fluoranthene (excitation 304 nm, emission 433 nm), and benzo(a)pyrene (excitation 380 nm, emission 406 nm). Identification of analytes was based on retention times and characteristic excitation/emission wavelength pairs. Quantification was carried out using external calibration with appropriate standard solutions. The limit of detection (LOD) for all polycyclic aromatic hydrocarbons (PAHs) was 0.5 µg/kg.

### 4.6. In Vitro Evaluation of Biochar Activity Using the Caco-2 Cell Model

The metabolic activity of Caco-2 was monitored using an MTS assay (Abcam, Cambridge, UK). The test was performed on semi-confluent Caco-2 cells (70–80% of confluency). Cells were seeded at a density of 1.2 × 10^3^ cells per well on a 96-well plate and cultured in 200 μL of CGM. Before the assay, cells were pre-cultured for 24 h in a CO_2_ incubator. Then, the complete growth medium (CGM) was replaced with BC-AE at concentrations of 0.5, 1, 2, 3, 4, 5, 6, 8, and 10 mg/mL. The metabolism of Caco-2 was determined after 24, 48, and 72 h of their propagation in the presence of BC-AE. The metabolic activity of cells was determined by adding 20 μL of MTS solution per well, followed by incubation with the dye for 2 h at 37 °C in a CO_2_ incubator. Absorbance was measured spectrophotometrically with a plate reader (Spark^®^, Tecan, Männedorf, Switzerland) at a wavelength of 490 nm. The acquired results were background-corrected and analyzed. All values were normalized to those of control cultures to determine the metabolic factor (MF), as described previously [45].

MF was calculated using the following formula:

$$MF=\frac{A_{490}\;treated\;sample-A_{490}\;blank}{A_{490}\;untreated\;sample-A_{490}\;blank}$$

The untreated control was assigned a value of 1.0, and all experimental values were expressed relative to this reference.

### 4.7. Functional In Vitro Assay—In-Depth Evaluation of BC-AE Influence on Caco-2 Viability

For the functional experiments, Caco-2 cells were inoculated at a density of 1.5 × 10^4^ cells/cm^2^. Based on a screening assay for functional analysis, the BC-AE was used at a concentration of 3 mg/mL, and cells were treated with BC-AE for 24 h and 72 h. Cells were cultured in the same manner as described above. The morphology of cells was monitored under an inverted microscope (Primo Vert, Zeiss, Oberkochen, Germany), equipped with an Axiocam 208 camera (Zeiss, Oberkochen, Germany) to evaluate changes in the growth pattern and morphology of Caco-2 cells treated with BC-AE. After the experiment, cells were harvested for molecular biology assays.

### 4.8. Evaluation of Gene Expression Patterns Modulated by BC-AE

The total RNA was isolated from Caco-2 cells using the phenol-chloroform method [48], applying previously described protocols [45]. RNA was isolated from 1 × 10^6^ cells, which were homogenized with 1 mL of TRI Reagent^®^ (Merck, Poznań, Poland). The quality and quantity of the isolated RNA were assessed spectrophotometrically using the DS-11 Fx DeNovix NanoSpectrophotometer (Wilmington, DE, USA). Before the reverse transcription, the RNA preparations (1 μg) were purified from genomic DNA by DNase I treatment (Thermo Fisher Scientific, Warsaw, Poland). The cDNA synthesis was performed using the Tetro cDNA Synthesis Kit (Meridian Bioscience Inc., Cincinnati, OH, USA) and the Mir-X™ miRNA First-Strand Synthesis Kit (Takara Clontech Laboratories, Biokom, Poznań, Poland) for gene expression and miRNA level detection, respectively. The RNA purification, gDNA elimination, and reverse transcription were carried out using a T100 Thermal Cycler (Bio-Rad, Hercules, CA, USA). The obtained cDNA was used as a template for quantitative PCR (qPCR), and transcript accumulation was monitored in real-time. The qPCRs were conducted with the SensiFast SYBR & Fluorescein Kit (Meridian Bioscience Inc., Cincinnati, OH, USA) on a CFX OPUS 384 System (Bio-Rad, Hercules, CA, USA). The total volume of the reaction mixture was 10 µL, the concentration of primers in each reaction was 400 nM (for mRNA detection) and 200 nM (for miRNA detection), while cDNA did not exceed 1% of the mix. The Cq values were normalized to ACTB and GAPDH for mRNA and U6 snRNA for miRNA. Average gene expression fold changes were calculated using the 2^−ΔΔCt^ method and RQ_MAX_ algorithm as described previously [49]. Primer sequences and details are provided in Supplementary data as Tables S2 and S3.

### 4.9. Evaluation of Cellular Morphology Modulated by BC-AE

To evaluate changes in Caco-2 morphology after BC-AE treatment, the experimental cultures were rinsed with HBSS and fixed using ice-cold 4% paraformaldehyde (PFA; Sigma-Aldrich/Merck Life Science Sp. z o.o., Poznan, Poland). The cultures were fixed for 15 min at room temperature, then left overnight at 4 °C to ensure complete fixation. Following fixation, the samples were carefully washed three times with HBSS containing 1% fetal bovine serum (FBS) to eliminate any remaining fixative. To facilitate intracellular staining, cell membranes were permeabilized by exposing the cultures to a 0.1% Triton X-100 solution prepared in HBSS for 15 min at room temperature. Cellular cytoskeletal actin filaments were stained with Atto-488-conjugated phalloidin (Sigma-Aldrich/Merck Life Science Sp. z o.o., Poznan, Poland) diluted 1:800 in HBSS. The cells were exposed to the dye for 40 min at room temperature in the dark. To visualize nuclei, Caco-2 cells were counterstained with 4′,6-diamidino-2-phenylindole dihydrochloride (DAPI) in mounting medium (ProLong™ Diamond Antifade Mountant with DAPI, Thermo Fisher Scientific, Warsaw, Poland). The protocol employed in this study was established in our earlier work [25]. The cell morphology and ultrastructure were analyzed using epifluorescence microscopy (Leica DMI8, Wetzlar, Germany) at 20-fold magnification, with images captured by a Leica K3M camera (Wetzlar, Germany). The analysis employed three fluorescence channels for (i) DAPI (excitation at 353 nm, emission at 465 nm), (ii) phalloidin (excitation at 493 nm, emission at 517 nm), and (iii) incorporated biomaterials (excitation at 590 nm, emission at 618 nm).

### 4.10. Evaluation of Apoptotic Activity Modulated by BC-AE

Apoptosis was assessed using flow cytometry with the Dead Cell Apoptosis Kit and Annexin V for Flow Cytometry (Thermo Fisher Scientific, Warsaw, Poland). A total of 1 × 10^5^ cells were harvested and stained according to the manufacturer’s instructions. Following staining, a minimum of 1 × 10^4^ cells per sample were analyzed on a CytoFLEX flow cytometer (Beckman Coulter, Brea, CA, USA). Annexin V-FITC and propidium iodide staining allowed for the clear differentiation of live, apoptotic, and necrotic cells. During the analysis debris was excluded based on forward scatter (FSC) and side scatter (SSC) characteristics, and the main cell population was selected for further analysis. Where applicable, doublets were excluded before apoptosis analysis. Annexin V-FITC and propidium iodide staining allowed the discrimination of viable (Annexin V−/PI−), early apoptotic (Annexin V+/PI−), late apoptotic/secondary necrotic (Annexin V+/PI+), and necrotic (Annexin V−/PI+) cells. Data acquisition and analysis were performed using CytExpert software (version 2.4, Beckman Coulter, CA, Brea, USA), with compensation and quadrant settings applied consistently across samples.

### 4.11. Evaluation of Protein Expression Modulated by BC-AE

Western blotting was performed following our previously established protocol [50] to identify intracellular proteins. After the experiment, Caco-2 cells were lysed in ice-cold RIPA buffer with 1% protease and phosphatase inhibitors. Protein concentrations were determined using the Pierce BCA Assay (Thermo Fisher Scientific, Warsaw, Poland). Samples were normalized to 10 µg protein, denatured at 95 °C for 5 min in 4× Laemmli buffer, and separated on 15% SDS-PAGE at 100 V for 90 min. Proteins were transferred onto a PVDF membrane (100 V, 60 min) and blocked with 5% skim milk in TBS-T (Bio-Rad, Warsaw, Poland). Membranes were incubated overnight at 4 °C with primary antibodies and then washed with TBS-T. Afterward, membranes were incubated with secondary antibodies for 60 min at room temperature and washed again. Chemiluminescent signals were detected using the Bio-Rad ChemiDoc™ XRS system with Pierce ECL substrate and analyzed with Image Lab™ Software (Version 6.1, Bio-Rad, Warsaw, Poland). Antibody details are provided in the Supplementary data as Table S4.

### 4.12. Statistical Analysis

The results are presented as means ± standard deviations (SD). All experiments were repeated three times independently (biological replicates, n = 3). Within each independent experiment, each condition was analyzed in at least three parallel wells (technical replicates). Statistical analysis was performed using mean values from independent biological replicates. Sample size was established using Stein’s two-stage procedure [51]. An initial pilot set of independent replicates was used to estimate variability, and the total number of replicates was then adjusted to achieve the minimum required sample size. Normality was tested with the Shapiro–Wilk test, and homogeneity of variances was assessed using Levene’s test, with the Brown–Forsythe test additionally applied when appropriate. Comparative statistical analyses were conducted using one-way analysis of variance (ANOVA), followed by Tukey’s post hoc test for multiple comparisons. This approach allowed pairwise comparisons among all analyzed groups, including both treated versus untreated control cultures and comparisons between different treatment conditions, while controlling the overall type I error rate. 6All statistical calculations were performed using GraphPad Prism version 10.0 (GraphPad Software, San Diego, CA, USA). Differences were considered statistically significant at *p* < 0.05.

## Data Availability

The Dataset is available on request from the authors.

## Supplementary Materials

The following supporting information can be downloaded at: https://www.mdpi.com/article/10.3390/molecules31060989/s1, Table S1. Multielemental analysis of biochar and biochar aqueous extract (BC-AE). Figure S1. FTIR spectra of biochar; Table S2. Primer sequences used for the detection of miRNA expression, including the characteristic, i.e., gene abbreviation, primer sequence (5′-3′), and annealing temperature; Table S3. Primer sequences used for the detection of mRNA expression, including the characteristic, i.e., transcript full name, gene abbreviation, primer sequence (5′-3′), locus, product length, annealing temperature, and reference number to the gene base; Table S4. List of primary antibodies used for protein detection, including protein name, abbreviation, dilution, product number, and supplier company.
