# Supplementary figures and images for: Evaluation of the Cytotoxicity of Biochar Aqueous Extract in Caco-2 Cells: Time-Dependent Regulation of Apoptosis, Associated with miRNA Modulation
Source: Molecules. 2026 Mar 16;31(6):989. doi: 10.3390/molecules31060989 (PMC13028679; doi:10.3390/molecules31060989)

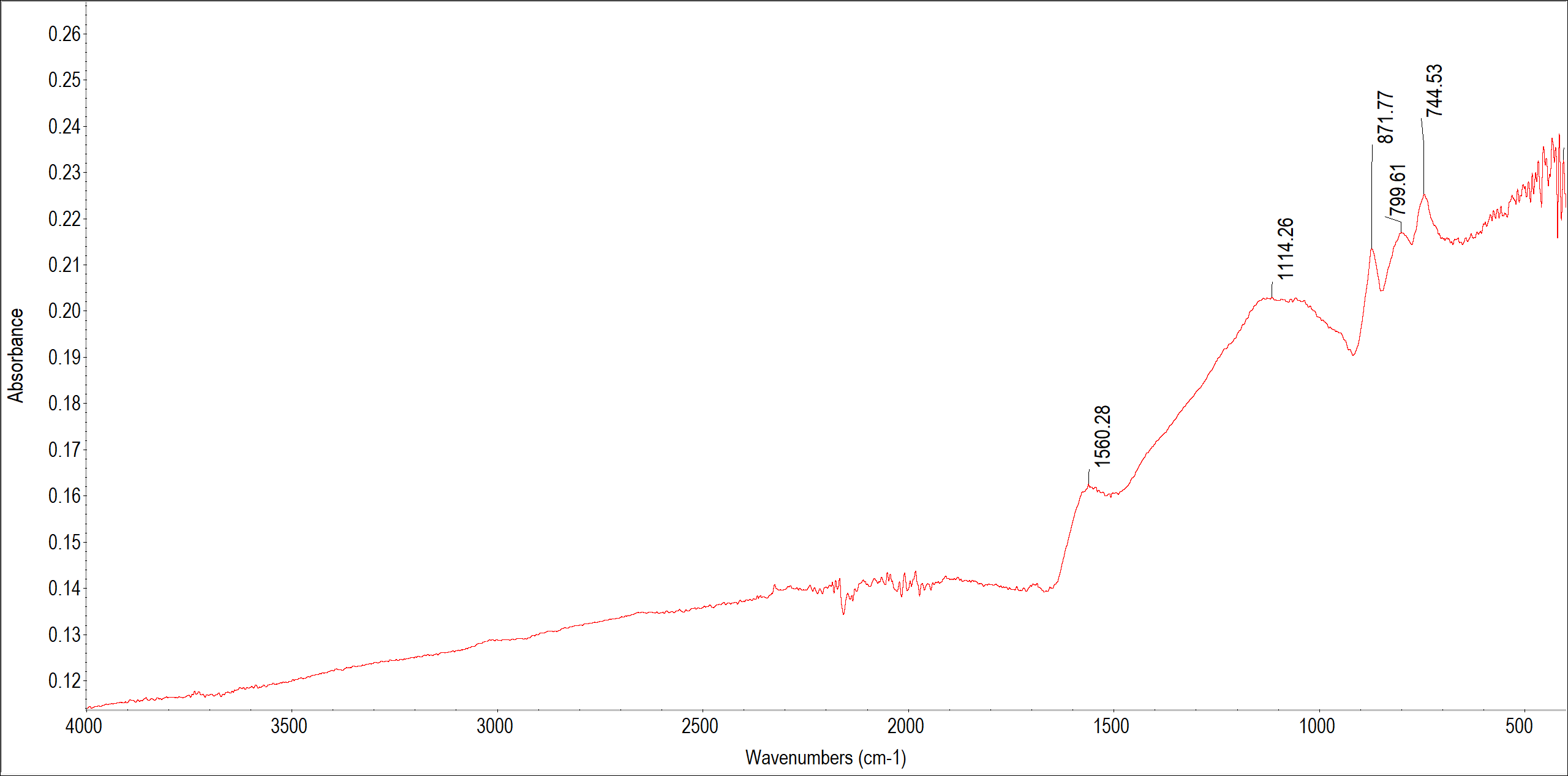

Supplement: Supplementary file 1 [file molecules-31-00989-s001.zip › Figure S1. FTIR spectra of biochar.TIF]
